# Real-world effectiveness of ixazomib, lenalidomide and dexamethasone in Asians with relapsed/refractory multiple myeloma

**DOI:** 10.1007/s12185-025-03927-z

**Published:** 2025-03-20

**Authors:** Soo Chin Ng, Joon-Ho Moon, Sung Soo Park, Youngil Koh, Ji Hyun Lee, Hyeon-Seok Eom, Ho-Jin Shin, Sung Hoon Jung, Young Rok Do, Gilbert Wilfred, Azlan Husin, Hyo Jung Kim, SFadilah Abdul Wahid, Myung-Won Lee, Hye-won Heo, Kihyun Kim, Suporn Chuncharunee

**Affiliations:** 1 Department of Haematology, Subang Jaya Medical Centre, Subang Jaya, Malaysia; 2 Department of Hematology-Oncology, Kyungpook National University Hospital, School of Medicine, Kyungpook National University, Daegu, Republic of Korea; 3 Department of Hematology, Seoul St. Mary’s Hospital, College of Medicine, The Catholic University of Korea, Seoul, Republic of Korea; 4 Department of Internal Medicine, Seoul National University Hospital, Seoul, Republic of Korea; 5 Department of Hematology-Oncology, Department of Internal Medicine, Dong-A University College of Medicine, Busan, Republic of Korea; 6 Center for Hematologic Malignancy, National Cancer Center, Goyang, Republic of Korea; 7 Department of Internal Medicine, Pusan National University Hospital, Pusan, Republic of Korea; 8 Department of Hematology-Oncology, Chonnam National University Hwasun Hospital, Hwasun, Republic of Korea; 9 Division of Hemato-Oncology, Keimyung University Dongsan Medical Center, Daegu, Republic of Korea; 10 Department of Medicine, Hospital Queen Elizabeth, Kota Kinabalu, Malaysia; 11 Department of Internal Medicine, Universiti Sains Malaysia, Gelugor, Malaysia; 12 Department of Internal Medicine, Hallym University Sacred Heart Hospital, Anyang, Republic of Korea; 13 Pusat Terapi Sel, Hospital Canselor Tuanku Muhriz, Kuala Lumpur, Malaysia; 14 Department of Internal Medicine, Chungnam National University Hospital, Daejeon, Republic of Korea; 15 Medical Affairs, Takeda Pharmaceuticals Korea Co. Ltd, Seoul, Republic of Korea; 16 Department of Medicine, Samsung Medical Center, Sungkyunkwan University School of Medicine, Seoul, Republic of Korea; 17 Department of Internal Medicine, Ramathibodi Hospital, Bangkok, Thailand

**Keywords:** Ixazomib, Real-world, Asia, Relapsed refractory multiple myeloma

## Abstract

Randomized clinical trials have shown ixazomib, lenalidomide and dexamethasone (IRd) to be efficacious and safe in Asian patients with relapsed/refractory multiple myeloma (RRMM); however, real-world data are limited. The APEX study was a multicenter, observational cohort study of IRd conducted at 16 sites across South Korea, Malaysia, and Thailand. Overall, 104 patients treated with IRd during 2016–2023 were enrolled; data were collected by retrospective chart review and 6-month prospective follow-up. Median age at IRd initiation was 64.0 years. The primary endpoints of median time to next treatment (TTNT) and overall response rate (ORR) were 32.1 months and 72.1%, respectively (though ORR varied across countries). The secondary endpoint of median progression-free survival was 27.7 months, while median overall survival was not reached. Median TTNT and ORR were higher in elderly patients (≥65 and/or ≥70 years) than in the overall population. Adverse events occurred in 90.4% and serious adverse events occurred in 29.8% of all patients; common Grade ≥ 3 adverse drug reactions were pneumonia (9.6%), neutropenia (7.7%), and gastroenteritis (2.9%). This study demonstrated that IRd was safe and effective in real-world practice in Asia, including for elderly patients, and the results are aligned with TOURMALINE-MM1 and other real-world studies.

**Supplementary Information:**

The online version contains supplementary material available at 10.1007/s12185-025-03927-z.

## Introduction

Multiple myeloma (MM) has a high prevalence worldwide, with over 130,000 cases reported in 2016; the highest number of cases were seen in Western Europe and North America [1]. However, the incidence of MM has been increasing in Asia in recent decades [1]. Over the past 15 years, novel treatments such as proteasome inhibitors and immunomodulatory drugs (IMiD) have been developed for MM, which have substantially improved survival outcomes for patients [2, 3]. Proteasome inhibitors (PIs) include bortezomib, carfilzomib and ixazomib [2, 4].

Ixazomib is a peptide boronic acid PI that is administered orally [3]. Ixazomib (I) was approved for use as a triplet regimen in combination with the IMiD lenalidomide (R) and steroid dexamethasone (d) [triplet combination—IRd] in the US in 2015, for patients with MM who have received at least one prior therapy [5]. This was based on the results of the global phase 3 study TOURMALINE-MM1, a double-blind and placebo controlled randomised trial, wherein treatment with IRd resulted in significantly longer median progression-free survival (PFS) versus placebo plus Rd (20.6 months vs. 14.7 months, respectively; HR for disease progression/death in the IRd group, 0.74; *P* = 0.01) [3]. The median overall survival (OS) for treatment with IRd was 53.6 months at the final analysis [6]. In the China continuation study of the TOURMALINE-MM1 trial, there was a 67% improvement in PFS with IRd vs. placebo-Rd (median PFS 6.7 months vs. 4.0 months, HR for disease/progression in the IRd group 0.598, *P* = 0.035), and the median OS for IRd at the final analysis was 25.8 months (vs. 15.8 months for placebo-Rd) [7].

Ixazomib became available in select countries in Asia via an Expanded Access Program (EAP) from 2016 onwards, which provided patients with life-threatening disease access to ixazomib for treatment outside of clinical trials, when no comparable or satisfactory alternative therapy options were available. However, there are limited real-world data on the effectiveness of IRd in Asian patients in clinical practice [4, 8], especially in trial-ineligible patient populations [9, 10]. Small studies from Korea and Japan [4, 8] have provided preliminary evidence that the effectiveness and safety of ixazomib in clinical practice is similar to results from the pivotal clinical trial, but it is necessary to understand whether this is concordant with other Asian countries. It would also be beneficial to understand whether the real-world findings from Asia reflect those of real-world studies for IRd in predominantly Western populations [11–14].

Thus, the Asia Pacific Real-world Experience of iXazomib (APEX) study investigated clinical characteristics, treatment patterns, and outcomes for patients with relapsed/refractory (RR) MM treated with IRd in the EAP program in Korea, Malaysia and Thailand with the aim to collect insights regarding clinical management and treatment gaps for patients with MM in Asia.

## Materials and methods

### Study design and patients

The APEX study was a multicentre, observational cohort study conducted in 16 sites specialised in the diagnosis and treatment of MM across three Asian countries: Korea, Malaysia, and Thailand. The study was conducted in two phases. In Phase 1, each site enrolled patients in the EAP who were treated with IRd between 2016–2023 and collected data retrospectively from patients’ existing medical charts. In Phase 2, prospective data were collected for a 6-month follow-up period for living patients, based on routine clinical practice.

The main eligibility criteria were as follows: All patients were adults aged ≥20 years with a confirmed diagnosis of MM, had received 1–3 prior lines of therapy, were of Eastern Cooperative Oncology Group (ECOG) performance status (PS) 0–2, were not refractory to lenalidomide/PIs, were in biochemical/symptomatic relapse and not on active anti-myeloma therapy for more than three cycles (except for steroids) at the time of ixazomib initiation, and had accessible medical records with details necessary for the study.

This study was conducted in accordance with ethical principles based on the Declaration of Helsinki, Good Clinical Practice (GCP), the International Society for Pharmacoepidemiology (ISPE) Guidelines for Good Pharmacoepidemiology Practice (GPP), and any applicable local regulations. The study was approved by the institutional review board (IRB) for each study site. The retrospective data collection did not require patient signatures on informed consent forms, in accordance with a waiver of requirement by the IRB. Written informed consent forms were signed by living patients or by the patients’ legally authorised representative before any prospective data were collected in the study database.

### Endpoints

The primary objective was to describe the effectiveness of IRd, from assessments of response based on the patients’ laboratory results and/or International Myeloma Working Group (IMWG) criteria of clinical practice guidelines [15]. The primary endpoints were time-to-next treatment (TTNT) and overall response rate (ORR). TTNT was defined as the time from initiation of IRd after registration for the EAP to the initiation of subsequent MM therapy; TTNT was censored for enrolled patients who were alive but did not receive subsequent therapy based on the date the patient’s information was last available, or for deceased patients who did not receive subsequent therapy based on the date of death. ORR was defined as the sum of stringent complete response (sCR), complete response (CR), very good partial response (VGPR), and partial response (PR) [15].

The secondary objectives were to describe the patient characteristics (demographics, clinical features at diagnosis, prior therapeutic regimens), and clinical outcomes (effectiveness and safety of IRd), including by age, line of treatment, previous treatment (PI, IMiD, R-exposure) and cytogenetic abnormality-based risk factors, as well as to evaluate the impact of patients’ characteristics on effectiveness and safety. Effectiveness was assessed by overall survival (OS), progression-free-survival (PFS), time-to-progression (TTP) and duration of treatment (DOT). OS was calculated as the time from initiation of IRd after registration for the EAP to the date of death (if death occurred). OS was censored for enrolled patients who were alive or lost to follow-up based on when the patient’s information was last available. PFS was calculated as the time from initiation of IRd after registration for the EAP to the date of first documentation of disease progression in medical charts or death from any cause, whichever came first. TTP was calculated as the time from initiation of IRd after registration for the EAP to the date of first documentation of disease progression in medical charts. PFS and TTP were censored for patients who started a subsequent MM therapy before documentation of progressive disease/death, based on the start date of subsequent MM therapy, and for those who were alive and without documentation of progressive disease/death based on when the patient’s information was last available. DOT was calculated as the time from the initiation of IRd after registration for the EAP to the end of IRd. Only treatment-emergent adverse events (TEAE) were analysed. In order to evaluate the impact of patient characteristics, clinical disease presentation and therapeutic regimens on IRd effectiveness, exploratory analyses were performed to assess predictive factors for TTNT and response.

### Data collection

The following information was extracted from patients’ institutional medical records:

- Population descriptors: Demographic characteristics, diagnostic and clinical characteristics, prior therapeutic regimens (type of therapy, line of treatment and response to prior therapy).
- Treatment: Exposure (to I, R and d) and outcome variables (effectiveness and safety of IRd overall by factors including line of treatment, previous therapies and cytogenetic abnormalities), response to IRd (date of response, disease progression), treatment for MM after IRd discontinuation (if applicable), survival, safety.

### Statistical analysis

No formal statistical hypotheses were set for this descriptive study, although a planned sample size of 136 patients was determined based on feasibility of data collection at the 16 sites. Statistical analyses of all data were performed using SAS^®^ statistical software 9.4 or higher (SAS Institute, Cary, NC, USA). The Kaplan–Meier method was used for analysis of TTNT, PFS, OS, DOT, TTP and time to response/best response for patients with ≥PR. The exploratory analysis of predictive factors for TTNT and response used Cox proportional hazard (PH) and logistic regression models, respectively, to assess each factor. Factors analysed in both the Cox PH and logistic regression models for TTNT and response, respectively, included gender, country of enrolment, comorbidities, ECOG performance status, cytogenetic risk, International Staging System (ISS) and Revised-ISS (R-ISS) stage, number of prior therapies, prior PI/IMiD therapy, relapsed or refractory MM and duration of ixazomib in IRd. Prior bortezomib or thalidomide response duration and R-exposure were also analysed in the Cox PH model for TTNT. The Cox PH model analysing prior bortezomib and thalidomide response duration and R-exposure was also used for PFS and OS.

## Results

### Baseline characteristics

**Fig. 1:**
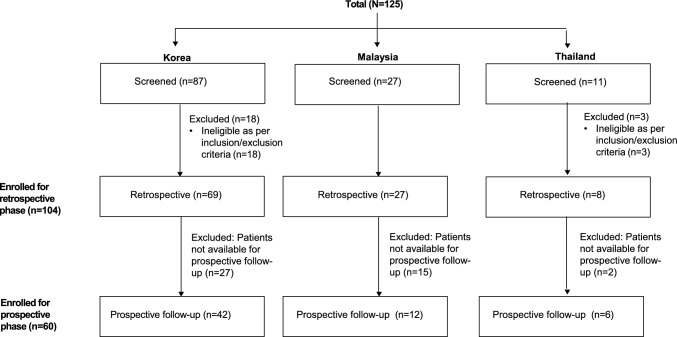
Patient disposition

A total of 125 patients (87 Korea, 27 Malaysia, 11 Thailand) provided informed consent for this study and underwent screening (Fig. 1). Of these, 21 patients (18 from Korea and three from Thailand) were excluded as they did not meet the eligibility criteria. Thus, the remaining 104 patients (69 from Korea, 27 from Malaysia, eight from Thailand) who fulfilled the eligibility criteria were enrolled in this study. Retrospective data were available for all 104 patients, while prospective data collection was performed for 60 patients (42 from Korea, 12 from Malaysia, six from Thailand).

Baseline characteristics of these patients are summarised in Table 1. Median age at treatment initiation was 64.0 years; 49% of patients were over 65 years of age and 31.7% of patients were over 70 years of age. Both genders were represented across participating countries. At diagnosis, more than half of all patients were classified as ISS stage II/III while over 48% of patients were classified as R-ISS stage II/III. Cytogenetics data were available for 60.6% of patients; 13.5% had high-risk cytogenetic abnormalities, of whom 8 had t(4;14), 5 had del(17p) and 1 had t(14;16).

**Table 1:** Patient demographics and baseline clinical characteristics

	Korea (*n* = 69)	Malaysia (*n* = 27)	Thailand (*n* = 8)	Total (*n* = 104)
*Patient demographics*				
Age (years), median^a^	67.0	60.0	62.0	64.0
≥65, *n* (%)	41 (59.4)	7 (25.9)	3 (37.5)	51 (49.0)
≥70, *n* (%)	27 (39.1)	4 (14.8)	2 (25.0)	33 (31.7)
Sex, male, *n* (%)	39 (56.5)	19 (70.4)	2 (25.0)	60 (57.7)
*Baseline clinical characteristics*				
ECOG performance status, *n* (%)				
0	19 (27.5)	2 (7.4)	5 (62.5)	26 (25.0)
1	36 (52.2)	15 (55.6)	1 (12.5)	52 (50.0)
2	3 (4.4)	8 (29.6)	1 (12.5)	12 (11.5)
3	2 (2.9)	1 (3.7)	1 (12.5)	4 (3.9)
Unknown	9 (13.0)	1 (3.7)	0 (0.0)	10 (9.6)
Type of multiple myeloma, *n* (%)				
IgG	28 (40.6)	16 (59.3)	6 (75.0)	50 (48.1)
Kappa	19 (27.5)	9 (33.3)	6 (75.0)	34 (32.7)
Lambda	9 (13.0)	7 (25.9)	0 (0.0)	16 (15.4)
IgA	11 (15.9)	6 (22.2)	2 (25.0)	19 (18.3)
Kappa	4 (5.8)	5 (18.5)	1 (12.5)	10 (9.6)
Lambda	7 (10.1)	1 (3.7)	1 (12.5)	9 (8.7)
Light chain only	28 (40.6)	3 (11.1)	0 (0.0)	31 (29.8)
Other^b^	2 (2.9)	2 (7.4)	0 (0.0)	4 (3.9)
ISS disease stage, *n* (%)				
I	19 (27.5)	2 (7.4)	0 (0.0)	21 (20.2)
II	12 (17.4)	6 (22.2)	5 (62.5)	23 (22.1)
III	28 (40.6)	6 (22.2)	0 (0.0)	34 (32.7)
Unknown	10 (14.5)	13 (48.2)	3 (37.5)	26 (25.0)
R-ISS disease stage, *n* (%)				
I	7 (10.1)	0 (0.0)	0 (0.0)	7 (6.7)
II	27 (39.1)	5 (18.5)	5 (62.5)	37 (35.6)
III	12 (17.4)	1 (3.7)	0 (0.0)	13 (12.5)
Unknown	23 (33.3)	21 (77.8)	3 (37.5)	47 (45.2)
Cytogenetic risk, *n* (%)				
High risk	12 (17.4)	1 (3.7)	1 (12.5)	14 (13.5)
t(4;14)	7 (10.1)	0 (0.0)	1 (12.5)	8 (7.7)
t(14;16)	1 (1.5)	0 (0.0)	0 (0.0)	1 (1.0)
del(17p)	4 (5.8)	1 (3.7)	0 (0.0)	5 (4.8)
Standard risk	38 (55.1)	6 (22.2)	5 (62.5)	49 (47.1)
Unknown	19 (27.54)	20 (74.07)	2 (25.00)	41 (39.42)
Renal function at IRd initiation (mL/min/1.73 m^2^), *n* (%)^a^				
eGFR ≥ 60	42 (60.9)	15 (55.6)	5 (62.5)	62 (59.6)
30 ≤ eGFR < 60	19 (27.5)	10 (37.0)	3 (37.5)	32 (30.8)
eGFR < 30	8 (11.6)	1 (3.7)	0 (0.0)	9 (8.7)
Unknown	0 (0.0)	1 (3.7)	0 (0.0)	1 (1.0)
*Disease status*				
Relapsed/Refractory multiple myeloma, *n* (%)^a^				
Relapsed	62 (89.9)	19 (70.4)	1 (12.5)	82 (78.9)
Refractory	5 (7.3)	0 (0.0)	4 (50.0)	9 (8.7)
Relapsed and refractory	2 (2.9)	8 (29.6)	3 (37.5)	13 (12.5)
Type of relapse, *n* (%)^a^				
Biochemical	53 (76.8)	20 (74.1)	3 (37.5)	76 (73.1)
Clinical	11 (15.9)	7 (25.9)	1 (12.5)	19 (18.3)

All values are at diagnosis of multiple myeloma, unless specified otherwise

*ECOG* Eastern Cooperative Oncology Group; *eGFR* estimated glomerular filtration rate; *Ig* immunoglobulin; *IRd* ixazomib, lenalidomide and dexamethasone; *ISS* International Staging System; *R-ISS* revised International Staging System

^a^Clinical characteristics at ixazomib-based regimen initiation

^b^1IgD lambda (Korea), 1 non-secretory (Malaysia), 2 unknown (1 Korea, 1 Malaysia)

At initiation of the ixazomib-based regimen, most patients had received one prior therapy (70.2%), although country-specific differences were seen (Table 2). Most patients from Korea had received one prior line of therapy (85.5% of patients), while most patients from Malaysia (55.6%) and Thailand (75.0%) had received 2 or 3 prior lines of therapy before initiating IRd. Notably, the median duration of prior therapies was much longer in Malaysia (41.0 months) versus Korea and Thailand (10.1 and 10.6 months, respectively). Overall, the median time from diagnosis to treatment with IRd was 36.3 months, but there was a wide range observed (2.5–177.7 months). Moreover, the median time from diagnosis to treatment with IRd was much longer in Malaysia (63.9 months) versus Korea (32.8 months) and Thailand (13.9 months). Almost half of all patients (48.1%) had received prior autologous stem cell transplant (SCT); no patient had received allogeneic SCT. The percentage of patients who received autologous SCT was highest in Malaysia (63.0%), versus 44.9% in Korea and 25.0% in Thailand. Over 90% of all patients had been treated with PIs; all patients had previously received bortezomib, and two patients had also received carfilzomib. Overall, 68.3% of patients had been treated with IMiDs—24.0% with lenalidomide and 58.7% with thalidomide. Lenalidomide was not used in Korea but was the only IMiD used in Thailand.

**Table 2:** Prior treatment

	Korea (*n* = 69)	Malaysia (*n* = 27)	Thailand (*n* = 8)	Total (*n* = 104)
Prior lines of therapy, *n* (%)^a^				
1	59 (85.5)	12 (44.4)	2 (25.0)	73 (70.2)
2	6 (8.7)	10 (37.0)	5 (62.5)	21 (20.2)
3	4 (5.8)	5 (18.5)	1 (12.5)	10 (9.6)
Duration of prior therapies (months), median (range)^a^	10.1 (0.3–106.2)	41.0 (6.0–104.6)	10.6 (3.0–54.2)	12.1 (0.3–106.2)
Time from diagnosis to ixazomib-based regimen (months), median (range)^a^	32.8 (5.6–177.7)	63.9 (12.1–116.4)	13.9 (2.5–94.3)	36.3 (2.5–177.7)
Prior SCT, *n* (%)^a^				
Autologous SCT	31 (44.9)	17 (63.0)	2 (25.0)	50 (48.1)
Prior proteasome inhibitor, *n* (%)^a^				
Yes	65 (94.2)	23 (85.2)	7 (87.5)	95 (91.4)
Bortezomib	65 (94.2)	23 (85.2)	7 (87.5)	95 (91.4)
Carflizomib	0 (0.0)	2 (7.4)	0 (0.0)	2 (1.9)
Prior immunomodulatory drug, *n* (%)^a^				
Yes	38 (55.1)	25 (92.6)	8 (100.0)	71 (68.3)
Lenalidomide	0 (0.0)	17 (63.0)	8 (100.0)	25 (24.0)
Thalidomide	38 (55.1)	23 (85.2)	0 (0.0)	61 (58.7)
Best response to most recent prior therapy, *n* (%)^a^				
sCR	3 (4.4)	5 (18.5)	0 (0.0)	8 (7.7)
CR	26 (37.7)	0 (0.0)	2 (25.0)	28 (26.9)
VGPR	16 (23.2)	9 (33.3)	0 (0.0)	25 (24.0)
PR	14 (20.3)	5 (18.5)	4 (50.0)	23 (22.1)

*CR* complete response, *PR* partial response, *sCR* stringent complete response, *SCT* stem cell transplant, *VGPR* very good partial response

^a^Clinical characteristics at ixazomib based regimen initiation

Best response to most recent prior therapy for ≥CR was 34.6%, although there were country-specific differences seen as ≥CR rates were lower in Malaysia (18.5%) and Thailand (25.0%) than in Korea (42.1%). The overall rates of PR and VGPR were 22.1% and 24.0%, respectively.

### Efficacy outcomes

**Fig. 2:**
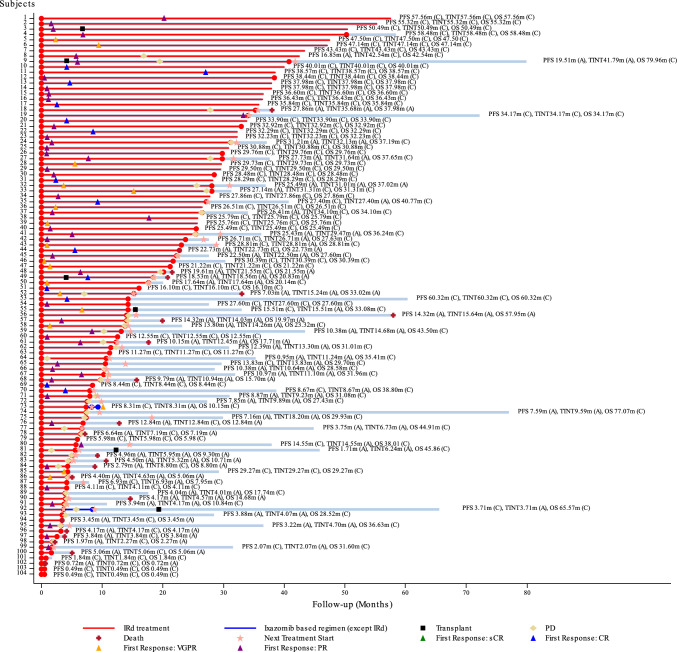
Swimmer plot for all patients. *A* actual event; *C* censored event; *CR* complete response; *IRd* ixazomib, lenalidomide and dexamethasone; *OS* overall survival; *PD* progressive disease; *PFS* progression free survival; *PR* partial response; *sCR* stringent complete response; *TTNT* time to next treatment; *VGPR* very good partial response

The swimmer plot for efficacy outcomes in all patients is shown in Fig. 2.

Median number of IRd cycles was 13.0 (range 1.0–50.0). However, the median number of IRd cycles in Thailand (42.0) was higher than in Korea (11.0) and Malaysia (13.0).

Primary outcomes are shown in Table 3. With a median follow-up duration of 29.7 months, median TTNT was 32.1 months (95% CI: [22.5, not reached (NR)]). The TTNT was higher in Korea (31.6 months [95% CI: 18.2, NR]) versus Malaysia (27.4 months [95% CI: 8.7, 41.8]), while the TTNT was not reached in Thailand. ORR was 72.1%, including VGPR or better in over 45% of patients; 27 patients achieved CR and one achieved stringent CR. ORR varied by country, with Korea and Thailand demonstrating a higher ORR than Malaysia (ORR for Malaysia was 48.2%, 95% CI [28.7, 68.1.]). The median time to response and the median time to the best response in patients who achieved a response of at least PR or better were 2.1 months (range 1.7–3.4 months) and 5.1 months (range 3.4–6.5 months), respectively.

**Table 3:** Primary outcomes for IRd

	Korea (*n* = 69)	Malaysia (*n* = 27)	Thailand (*n* = 8)	Total (*n* = 104)
Follow-up duration (months), median (range)	28.5 (0.7–38.4)	38.8 (0.5–80.0)	47.3 (20.8–58.5)	29.7 (0.5–80.0)
TTNT (months), median (95% CI)^a^	31.6 (18.2, NR)	27.4 (8.7, 41.8)	NR (6.2, NR)	32.1 (22.5, NR)
*Best response, n (%) [95% CI]*				
sCR	1 (1.5) [0.0, 7.8]	0 (0.0) [0.0, 12.8]	0 (0.0) [0.0, 36.9]	1 (0.96) [0.02, 5.24]
CR	18 (26.1) [16.3, 38.1]	5 (18.5) [6.3, 38.1]	4 (50.0) [15.7, 84.3]	27 (26.0) [17.9, 35.5]
VGPR	14 (20.3) [11.6, 31.7]	2 (7.4) [0.9, 24.3]	3 (37.5) [8.5, 75.5]	19 (18.3) [11.4, 27.1]
PR	22 (31.9) [21.2, 44.2]	6 (22.2) [8.6, 42.3]	0 (0.0) [0.0, 36.9]	28 (26.9) [18.7, 36.5]
MR	3 (4.4) [0.9, 12.2]	2 (7.4) [0.9, 24.3]	0 (0.0) [0.0, 36.9]	5 (4.8) [1.6, 10.9]
SD	6 (8.7) [3.3, 18.0]	3 (11.1) [2.4, 29.2]	0 (0.0) [0.0, 36.9]	9 (8.7) [4.0, 15.8]
PD	4 (5.8) [1.6, 14.2]	3 (11.1) [2.4, 29.2]	1 (12.5) [0.3, 52.7]	8 (7.7) [3.4, 14.6]
ORR, *n* (%) [95% CI]	55 (79.7) [68.3, 88.4]	13 (48.2) [28.7, 68.1]	7 (87.5) [47.4, 99.7]	75 (72.1) [62.5, 80.5]
Time to response^b^ in ≥PR patients (months), median (95% CI)^a^	1.8 (1.2, 2.1)	6.4 (4.2, 9.3)	2.4 (1.5, 7.7)	2.1 (1.7, 3.4)
Time to best response^c^ in ≥PR patients (months), median (95% CI)^a^	3.4 (2.0, 5.3)	6.5 (4.2, 9.3)	16.9 (5.9, NR)	5.1 (3.4, 6.5)

*CI* confidence interval; *CR* complete response; *IRd* ixazomib, lenalidomide and dexamethasone; *MR* minimal response; *NR* not reached; *ORR* overall response rate; *PD* progressive disease; *PR* partial response; *sCR* stringent complete response; *SD* stable disease; *TTNT* time to next treatment; *VGPR* very good partial response

^a^Kaplan–Meier method

^b^From the first date of IRd to the date of PR achievement

^c^From the first date of IRd to the date of best response achievement

**Fig. 3:**
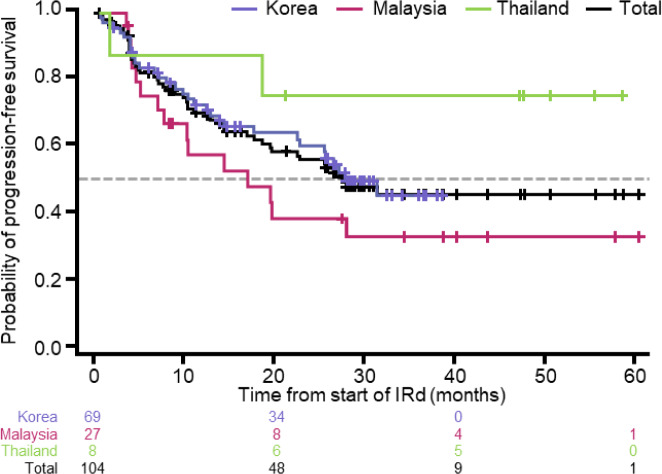
Kaplan–Meier analysis of progression-free survival. *IRd* ixazomib, lenalidomide and dexamethasone

Secondary outcomes are shown in Table 4. The median duration of treatment was 14.8 months (95% CI [11.3, 23.0]). Median PFS was 27.7 months (95% CI [19.5–NR]) (Fig. 3), while the median OS was not reached. The median time to progression was 31.2 months (95% CI [25.4, NR]), but varied between countries.

**Table 4:** Secondary outcomes: survival analysis of DOT, OS, PFS, and TTP for IRd

	Korea (*n* = 69)	Malaysia (*n* = 27)	Thailand (*n* = 8)	Total (*n* = 104)
DOT (months), median (95% CI)^a^	14.3 (10.4, 25.4)	13.2 (4.5, 27.1)	47.3 (5.6, 50.5)	14.8 (11.3, 23.0)
OS (months), median (95% CI)^a^	NR (NR, NR)	NR (33.0, NR)	NR (20.8, NR)	NR (58.0, NR)
PFS (months), median (95% CI)^a^	27.7 (22.5, NR)	16.9 (7.0, NR)	NR (1.7, NR)	27.7 (19.5, NR)
TTP (months), median (95% CI)^a^	31.2 (25.4, NR)	19.5 (10.2, NR)	NR (1.7, NR)	31.2 (25.4, NR)

*CI* confidence interval; *DOT* duration of treatment; *IRd* ixazomib, lenalidomide and dexamethasone; *NR* not reached; *OS* overall survival; *PFS* progression-free survival; *TTP* time to progression

^a^Kaplan–Meier method

Sub-group analyses were performed to determine if the primary outcomes (ORR and TTNT) differed by prior SCT, PI or IMiDs, age at initiation of IRd, and cytogenetic risk (the latter determined at MM diagnosis) (Table 5). Interestingly, in elderly patients ≥65 years (49.0% of patients), median TTNT was 35.7 months (95% CI [28.8, NR]) and ORR was 80.4% (95% CI [66.9, 90.2]), which was higher than that of patients <65 years (median TTNT 22.5 months [95% CI, (12.5, NR)] and ORR 64.2% [95% CI, 49.8, 76.9]). Similarly, in elderly patients ≥70 years (31.7% of patients), median TTNT was also 35.7 months (95% CI [18.2, NR]) and ORR was 81.8% (95% CI [64.5, 93.0]), which was higher than that of patients <70 years (median TTNT 29.5 months [95% CI, (14.6, NR)] and ORR 67.6% [95% CI, 55.5, 78.2]). For patients with high-risk cytogenetics, the median TTNT was 14.3 months (95% CI [6.2, NR]), which was lower than that of patients with standard cytogenetic risk (median TTNT 35.7 [95% CI, 17.6, NR]), although ORR was only slightly lower than that of patients with standard cytogenetic risk (71.4% versus 77.6%, respectively). Patients with no prior SCT had a higher TTNT (35.7 months, 95% CI [18.2, NR]) and ORR (77.8%, 95% CI [64.4, 88.0]) than patients who had been treated with SCT previously (median TTNT 29.5 months [95% CI, 14.7, NR] and ORR 66.0% [95% CI, 51.2, 78.8]). However, patients treated with prior PIs had a slightly higher ORR (72.6% [95% CI, 62.5, 81.3]) versus those who had not received prior PI therapy (66.7% [95% CI, 29.9, 92.5]). For prior IMiDs, the subgroup with no prior IMiD had higher ORR (78.8%, 95% CI [61.1, 91.0]) than the group which had prior IMiD (69.0, 95% CI [56.9, 79.5]). Similarly, patients with no R-exposure had a higher ORR than those with R-exposure (74.7% versus 64.0%, respectively). Of note, the median R-exposure duration was 314 days (range 21–1237) for the R-exposure group. The PFS was NR (95% CI [14.3, NR]) for the R-exposure group whereas the PFS was 26.4 months (95% CI, 14.3, NR) for the R-naïve group.

**Table 5:** Subgroup analysis of TTNT and ORR

	Korea (*n* = 69)	Malaysia (*n* = 27)	Thailand (*n* = 8)	Total (*n* = 104)
*TTNT (months), median (95% CI)*^a^				
Age at initiation of ixazomib-based regimen (years)				
<65	15.5 (10.6, NR)	15.6 (8.3, 41.8)	NR (6.2, NR)	22.5 (12.5, NR)
≥65	31.6 (28.8, NR)	NR (6.7, NR)	NR (18.6, NR)	35.7 (28.8, NR)
<70	29.5 (14.3, NR)	15.6 (8.3, 41.8)	NR (6.2, NR)	29.5 (14.6, NR)
≥70	31.6 (17.6, NR)	NR (15.2, NR)	NR (NR, NR)	35.7 (18.2, NR)
Cytogenetic risk^b^				
Standard	32.1 (18.2, NR)	12.1 (5.3, NR)	NR (NR, NR)	35.7 (17.6, NR)
High risk	20.6 (5.7, NR)	8.7 (NR, NR)	6.2 (NR, NR)	14.3 (6.2, NR)
Prior stem cell transplant				
Yes	NR (15.5, NR)	14.7 (6.7, 34.2)	NR (6.2, NR)	29.5 (14.7, NR)
No	31.0 (13.8, NR)	NR (4.0, NR)	NR (18.6, NR)	35.7 (18.2, NR)
Prior proteasome inhibitor therapy				
Yes	31.0 (17.6, NR)	15.6 (8.7, 41.8)	NR (6.24, NR)	31.0 (18.2, NR)
No	NR (NR, NR)	34.2 (8.3, NR)	NR (NR.NR)	NR (8.3, NR)
Prior IMiD				
Yes	28.8 (14.3, NR)	15.6 (8.7, 41.8)	NR (6.2, NR)	29.5 (15.2, NR)
No	32.1 (17.6, NR)	NR (NR, NR)	NR (NR, NR)	32.1 (17.6, NR)
*ORR, n/N (%) [95% CI]*				
Age at initiation of ixazomib-based regimen (years)				
<65	20/28 (71.4) [51.3, 86.8]	10/20 (50.0) [27.2, 72.8]	4/5 (80.0) [28.4, 99.5]	34/53 (64.2) [49.8, 76.9]
≥65	35/41 (85.4) [70.8, 94.4]	3/7 (42.9) [9.9, 81.6]	3/3 (100.0) [29.2, 100.0]	41/51 (80.4) [66.9, 90.2]
<70	33/42 (78.6) [63.2, 89.7]	10/23 (43.5) [23.2, 65.5]	5/6 (83.3) [35.9, 99.6]	48/71 (67.6) [55.5, 78.2]
≥70	22/27 (81.5) [61.9, 93.7]	3/4 (75.0) [19.4, 99.4]	2/2 (100.0) [15.8, 100.0]	27/33 (81.8) [64.5, 93.0]
Cytogenetic risk^b^				
Standard	31/38 (81.6) [65.7, 92.3]	2/6 (33.3) [4.3, 77.7]	5/5 (100.0) [47.8, 100.0]	38/49 (77.6) [63.4, 88.2]
High risk	9/12 (75.0) [42.8, 94.5]	1/1 (100.0) [2.5, 100.0]	0/1 (0.0) [0.0, 97.5]	10/14 (71.4) [41.9, 91.6]
Prior stem cell transplant				
Yes	25/31 (80.7) [62.5, 92.6]	7/17 (41.2) [18.4, 67.1]	1/2 (50.0) [1.3, 98.7]	33/50 (66.0) [51.2, 78.8]
No	30/38 (79.0) [62.7, 90.5]	6/10 (60.0) [26.2, 87.8]	6/6 (100.0) [54.1, 100.0]	42/54 (77.8) [64.4, 88.0]
Prior proteasome inhibitor therapy				
Yes	52/65 (80.0) [68.2, 88.9]	11/23 (47.8) [26.8, 69.4]	6/7 (85.7) [42.1, 99.6]	69/95 (72.6) [62.5, 81.3]
No	3/4 (75.0) [19.4, 99.4]	2/4 (50.0) [6.8, 93.2]	1/1 (100.0) [2.5, 100.0]	6/9 (66.7) [29.9, 92.5]
Prior IMiD				
Yes	30/38 (79.0) [62.7, 90.5]	12/25 (48.0) [27.8, 68.7]	7/8 (87.5) [47.4, 99.7]	49/71 (69.0) [56.9, 79.5]
No	25/31 (80.7) [62.5, 92.6]	1/2 (50.0) [1.3, 98.7]	NE [NE]	26/33 (78.8) [61.1, 91.0]
R-exposure				
Yes	–	–	–	16/25 (64.0) [42.5, 82.0]
No	–	–	–	59/79 (74.7) [63.6, 83.8]

*CI* confidence interval, *IMiD* immunomodulatory drug, *ORR* overall response rate, *NE* not estimable, *NR* not reached, *R* lenalidomide, *TTNT* time to next treatment

^a^Kaplan–Meier method

^b^Analysed based on clinical characteristics at multiple myeloma diagnosis

Exploratory analyses examining patient factors associated with TTNT indicated that none of the patient characteristics or demographic/disease-related factors explored, including age (≥65 years), gender, ECOG performance status, ISS/R-ISS, high-risk cytogenetics, number of prior lines of therapy, prior proteasome inhibitor, prior IMiD/R-exposure, relapsed/refractory MM, prior bortezomib or thalidomide response duration or country of enrolment were predictive of TTNT; *P* values for all associations were >0.05 in univariate cox regression analyses (Supplementary Table 1). However, in exploratory analyses using the same factors for ORR, country of enrolment was identified as a significant predictive factor (*P* = 0.007) (Supplementary Table 2). No predictive factors for PFS were identified in the univariate Cox regression analysis, which included prior bortezomib and thalidomide response duration, and R-exposure as covariates (*P* > 0.05) (Supplementary Table 3). However, in the univariate Cox regression analysis of OS, prior bortezomib response duration of 12 months (*P* = 0.007) and prior thalidomide response duration of 12 months (*P* = 0.007) and 18 months (*P* = 0.003) were identified as significant predictive factors of OS (Supplementary Table 4).

### Safety

Safety outcomes are shown in Table 6. AEs occurred in 94 patients (90.4%) and serious AEs in 31 patients (29.8%). Adverse drug reactions (ADRs) occurred in 77 patients (74.0%).

**Table 6:** Safety outcomes

	Korea (*n* = 69)	Malaysia (*n* = 27)	Thailand (*n* = 8)	Total (*n* = 104)
AEs, *n* (%)				
Any grade^a^	62 (89.9)	25 (92.6)	7 (87.5)	94 (90.4)
Serious AE^b^	13 (18.8)	17 (63.0)	1 (12.5)	31 (29.8)
Adverse drug reactions^a^, *n* (%)				
Any grade	51 (73.9)	20 (74.1)	6 (75.0)	77 (74.0)
Grade ≥ 3	–	–	–	29 (27.9)
Select ADRs (Grade ≥ 3)^c^				
Pneumonia	–	–	–	10 (9.6)
Neutropenia	–	–	–	8 (7.7)
Gastroenteritis	–	–	–	3 (2.9)
Reduction of the first administration dosage for IRd regimen, *n* (%)				
Ixazomib	21 (30.4)	2 (7.4)	0 (0.0)	23 (22.1)
Lenalidomide	34 (49.3)	1 (3.7)	3 (37.5)	38 (36.5)
Dexamethasone	32 (46.4)	3 (11.1)	1 (12.5)	36 (34.6)
Relative dose intensity for IRd regimen, mean ± standard deviation^d^				
Ixazomib	0.9 ± 0.2	0.9 ± 0.1	1.0 ± 0.0	0.9 ± 0.1
Lenalidomide	0.6 ± 0.3	0.7 ± 0.3	0.8 ± 0.2	0.7 ± 0.3
Dexamethasone	0.6 ± 0.3	0.6 ± 0.3	0.8 ± 0.3	0.6 ± 0.3
AEs resulting in discontinuation, *n* (%)	15 (21.7)	3 (11.1)	1 (12.5)	19 (18.3)
AEs leading to death, *n* (%)	3 (4.4)	1 (3.7)	0 (0.0)	4 (3.9)

*ADR* adverse drug reaction; *AE* adverse events; *IRd* ixazomib, lenalidomide and dexamethasone

^a^Occurring after IRd/ixazomib based regimen initiation

^b^Considered “serious” if it is any untoward medical occurrence at any dose: results in death; in the view of the healthcare provider, places the patient at immediate risk of death, although this does not include an event that, had it occurred in a more severe form, might have caused death; requires inpatient hospitalisation or prolongation of existing hospitalisation; results in persistent or significant disability/incapacity; results in a congenital anomaly/birth defect; it may also be any other medically important event that, in the opinion of the healthcare provider, may jeopardise the patient or may require intervention to prevent one of the other outcomes

^c^MedDRA (version 26.0)

^d^Relative dose intensity was calculated from all subjects and planned dose was calculated by standard dose (ixazomib: 4 mg)

Non-haematologic ADRs were more common than haematologic ADRs. Amongst the non-haematological ADRs, gastrointestinal toxicities were the most common; diarrhoea, nausea and constipation occurred in 15, 8 and 6 patients, respectively. Infections or infestations were reported in 22 patients, of whom 12 patients had pneumonia. Nervous system disorders were also common; 11 patients had peripheral neuropathy while 5 patients had peripheral sensory neuropathy (15 of the 16 patients with these nervous system disorders were from Korea). Haematological ADRs of neutropenia and thrombocytopenia occurred in 8 (7.7%) and 5 (4.8%) patients, respectively. Diarrhoea was a common ADR in all three countries (14.5% in Korea, 14.8% in Malaysia and 12.5% in Thailand). The other common ADRs differed based on the country of enrolment; peripheral neuropathy (14.5%) and asthenia (11.6%) were common in Korea, while pneumonia (22.2%) and anaemia (11.1%) were common in Malaysia. One patient each (12.5%) had pneumonia, gingival abscess, peripheral neuropathy, neutropenia, leukopenia and pancytopenia in Thailand.

Grade ≥ 3 ADRs occurred in 29 patients (27.9%). The most common Grade ≥ 3 ADRs were pneumonia (9.6%), neutropenia (7.7%), and gastroenteritis (2.9%).

At first administration of IRd, dose reduction for ixazomib was only required in 22.1% patients versus 36.5% for lenalidomide and 34.6% for dexamethasone. The proportion of patients who required dose reductions was higher in Korea for all three drugs, compared with Malaysia and Thailand. The main reason for dose modification was toxicities (Table 6). The mean relative dose intensity (RDI) for ixazomib was 0.9 ± 0.1, which was higher than for lenalidomide (0.7 ± 0.3) and dexamethasone (0.6 ± 0.3). Mean RDI for all three drugs was higher in Thailand than in Korea and Malaysia. The main reasons for discontinuation were disease progression and toxicity. Overall, 19 patients (18.3%) discontinued due to AEs, including constipation, nausea and pneumonia (2% each). Four patients (3.9%) died due to AEs, three due to pneumonia (2.9%) and one due to sepsis (1.0%).

## Discussion

The APEX real-world study for IRd in Asia–Pacific demonstrated the effectiveness and manageable safety profile of IRd in Asian patients with RRMM.

The median PFS (27.7 months) was observed to be longer in the APEX study than in the TOURMALINE-MM1 trial (20.6 months), although the studies are not directly comparable [3]. The median PFS was also higher than that observed for IRd in previous real-world studies in Asia (25.9 months in a Korean study and 15.3 months in a Japanese study) [4, 8]. Notably, the median PFS was higher than the PFS observed in other real-world studies conducted primarily in Western countries, including patients from INSIGHT-MM and the Czech Registry of Monoclonal Gammopathies (RMG) [median PFS 21.2 months], Czech RMG alone [median PFS 17.5 months] and from Greece, UK and the Czech Named Patient Program (NPP) [median PFS 27.6 months] [11–13]. The higher PFS reported in the APEX study may be due to the lower number of prior lines of therapy in this study (1–3, with most patients receiving one prior therapy) compared to patients in the other real-world studies who had received up to nine lines of therapy [11–14]. Moreover, PI and lenalidomide-refractory patients were excluded from the study, unlike other real-world studies [16]. The INSURE real-world analysis has shown that patients who are refractory to PIs or lenalidomide in previous lines of therapy do not have equivalent TTNT and ORR benefits when compared with patients who are not refractory to thesethese agents [16, 17]. However, when treating patients with RRMM in routine clinical practice, prior exposure to lenalidomide or a PI should not preclude the use of IRd in subsequent lines of therapy [17].

The ORR of IRd in the APEX study (72.1%) was aligned with the ORR observed in TOURMALINE-MM1 (78.0%) [3] and other real-world studies highlighted above (54–85%) [4, 8, 11–13]. However, the ORR for IRd varied between countries in the APEX study: ORR was lower in Malaysia than in Korea and Thailand. Country of enrolment was identified as a significant predictive factor in the exploratory analysis as well. A potential reason for this difference could be the much longer median time from diagnosis to IRd use in Malaysia, as well as the longer duration of prior therapies in Malaysia. It is possible that the length of pre-treatment with alternative therapies affected the overall response to IRd, and the PFS and TTP (both median PFS and TTP were shorter in Malaysia than in Korea). Moreover, one-third of the patients recruited from Malaysia were of ECOG PS 2–3 versus only 7.3% of Korean patients and a quarter of the patients from Thailand. In previous real-world analyses, IRd has demonstrated greater benefit in non-frail patients versus frail patients and ECOG PS 0–1 has been associated with a lower risk of progression or death [13, 16, 18]. Although IMWG Frailty Score data were not collected in this retrospective chart review, because Frailty Score is not commonly calculated in real world practice in Asia, further investigation in future research studies may be warranted to assess any differences in frailty between patient populations in different countries.

Notably, in the sub-group analysis, the benefit of IRd was seen in elderly patients (≥65 and ≥70 years); the sub-group for ≥65 years also included the ≥70-year age group (no specific analyses were conducted comparing the ≥65 and ≥70-year-old age groups). In fact, TTNT was longer and ORR was higher in elderly patients than in the overall population, and in patients <65/70 years of age. This indicates that IRd is effective in elderly patients, who typically have poor prognosis, and is consistent with results from other real-world studies such as US MM-6 [19] and REMIX [18]. This also corroborates the results of an Italian real-world multicentre study which demonstrated that IRd leads to favourable PFS outcomes in patients ≥75 years [20]. Though consistent ORR benefits were seen in the subgroup with high-risk cytogenetics in this study, the TTNT was lower than that for the subgroup with standard risk. Although the TOURMALINE MM1 trial and a real-world study conducted in Israel showed that cytogenetic risk did not affect PFS outcomes for IRd treatment [3, 21], and an analysis of electronic health records from the US also demonstrated that IRd tends to produce better outcomes than bortezomib-Rd and carfilzomib-Rd in patients with high-risk cytogenetics [22]; further studies are therefore needed to support these findings. Also, although prior SCT and R-exposure led to slightly lower ORR (as expected based on observations regarding R-exposure from a previous real-world study) [12], almost two thirds of patients in the APEX study responded (ORR 64–66%). Previously, the Czech RMG real-world study has also shown that prior SCT leads to increased median PFS in the IRd group [14], while the INSURE study demonstrated that prior R-exposure does not affect IRd effectiveness [16, 17]. More real-world studies would be required to fully understand the effect of prior therapy on effectiveness of IRd. Thus, this study demonstrates that IRd is an effective treatment in real-world practice in Asia, especially in elderly patients.

The safety profile of IRd observed in this study was also manageable. Only 22.1% of patients required dose modification of the first dose of ixazomib, which is similar to the 17% who required dose modifications in the real-world analyses for INSIGHT MM and Czech RMG [11]. A lower proportion of patients required ixazomib dose reductions versus lenalidomide (similar to the trend observed in the previous real-world study in Korea [4]) and dexamethasone, and RDI was consequently higher for ixazomib versus the other two agents. Interestingly, Korea had the highest dose reductions for all three agents, compared to Malaysia and Thailand. However, the RDI for ixazomib was similar for Korea and Malaysia, while patients in Thailand maintained their initial dose of ixazomib. Of note, serious AEs were observed in only 29.8% of patients in the APEX study, versus in 47.0% patients in TOURMALINE-MM1, 37.8% of patients in the REMIX study and in 40.0% of the patients in the previous real-world study of IRd in Korea [3, 4, 16]. The rate of serious haematological toxicities was low, as was the frequency of other serious ADRs (pneumonia and gastrointestinal disorders). Notably, Grade ≥ 3 ADR neutropenia occurred in only 7.7% of patients in the APEX study compared with 23% of patients in the TOURMALINE-MM1 trial and 12% of patients in the previous real-world study conducted in Korea [3, 4]. Though diarrhoea (any grade ADR) was common in all three countries (12.5–14.8%), the frequency was similar to the REMIX study (13.9%) and lower than the AE rate of diarrhoea (any grade) observed in TOURMALINE-MM1 (45%), Czech RMG (34.7%) and the previous Korean real-world study (22%) [3, 4, 12, 18].

This study also highlights country-specific differences and treatment patterns for RRMM within Asia. The proportion of patients who were elderly was higher in Korea (59.4% ≥65 years in Korea, versus 25.9% in Malaysia and 37.5% in Thailand). Interestingly, most patients from Korea had received only one prior line of therapy (85.5%) and had a shorter median duration of prior therapies (10.1 months), while most patients from Malaysia (55.6%) had received 2 or 3 prior lines of therapy before initiating IRd and had a much longer median duration of prior therapies (41.0 months); most patients in Thailand (75.0%) had also received 2 or 3 prior lines of therapy but for a much shorter median duration (10.6 months). Consequently, patients from Malaysia typically had higher ECOG-PS and had a much longer median time from diagnosis to initiation of IRd than those observed in Korea and Thailand, which may have influenced the difference in effectiveness outcomes for Malaysia. SCT was the most common early treatment in Malaysia (63.0% of patients) versus its use for 45.0% of patients in Korea and only 25.0% of patients in Thailand. It is interesting to note that most patients had received prior PI therapy with bortezomib, irrespective of their country of enrolment. Similar to observations from other real-world studies [12, 14], these patients still derived clinical benefit from IRd. Carflizomib was used to treat only two patients in Malaysia prior to initiating IRd. Prior IMiDs were common for most patients in Malaysia and Thailand (92.6% and all patients, respectively) but only 55.1% of patients in Korea. Interestingly, all patients in Korea received thalidomide and all patients in Thailand were treated with lenalidomide, while both thalidomide and lenalidomide were used in Malaysia. CR or sCR to the most recent prior therapy was higher in Korea (over 40.0% of patients with CR or sCR, most of whom had received bortezomib) versus 18.5% of patients in Malaysia and 25.0% of patients in Thailand. Further data using larger datasets would be necessary to better understand any country-specific differences or genetic influences in toxicities and effectiveness affecting long-term use of these PIs.

As with any real-world study, there are several limitations of this study, including the unavailability of all relevant data in patient medical records based on clinical practice in each country or study site, diagnosis of RRMM and disease progression ascertained only by the treating centre (not by a central review committee), as well as the possibility of missing data that might confound the results. To address the issue of missing data regarding disease progression, both TTNT and PFS were assessed, since TTNT can be a proxy for PFS when there is missing information regarding disease progression [16]. Moreover, censoring criteria were adjusted to accommodate for the challenges of data collection in the real world. However, the main limitation of this study was the small number of participants from each country, which also limits interpretation of results from the subgroup analyses and the validity of comparing national trends.

In conclusion, the APEX study demonstrates that IRd is effective in the real-world setting in Asia, with a manageable safety profile. ORR of IRd in the APEX study was aligned with that in TOURMALINE-MM1 and other real-world studies of RRMM, while the median PFS was longer than in TOURMALINE-MM1 (though country-specific differences were seen, and these studies are not directly comparable) [3]. IRd is effective for a broad range of patients with RRMM, including elderly patients, who typically have poor prognosis.

## Supplementary Information

Below is the link to the electronic supplementary material.

Supplementary file1 (DOCX 62 KB)

## Data Availability

The datasets, including the redacted study protocol, redacted statistical analysis plan, and individual participants data supporting the results reported in this article, will be made available within 3 months from initial request to researchers who provide a methodologically sound proposal. The data will be provided after its de-identification, in compliance with applicable privacy laws, data protection and requirements for consent and anonymisation.
