# Supplementary material for: Real-world effectiveness of ixazomib, lenalidomide and dexamethasone in Asians with relapsed/refractory multiple myeloma
Source: Int J Hematol. 2025 Mar 20;121(5):670–83. doi: 10.1007/s12185-025-03927-z (PMC12014722; doi:10.1007/s12185-025-03927-z)
Supplement: Supplementary file 1 — Supplementary file1 (DOCX 62 KB) [file 12185_2025_3927_MOESM1_ESM.docx]

**SUPPLEMENT:** Exploratory analysis of patient characteristics associated with efficacy outcomes in the APEX study

**Supplementary Table 1** Univariate Cox regression analysis of factors associated with time to next treatment

|  | | N | # of events | Hazard Ratio | 95% CI | P-value |
| --- | --- | --- | --- | --- | --- | --- |
| Age (years) | <65 | 53 | 27 | 1 | Reference | 0.084 |
|  | ≥65 | 51 | 19 | 0.60 | [0.33, 1.07] |  |
| Gender | Male | 60 | 30 | 1 | Reference | 0.332 |
|  | Female | 44 | 16 | 0.74 | [0.40, 1.36] |  |
| Country of enrolment | Korea | 69 | 29 | 1 | Reference | 0.195 |
|  | Malaysia | 27 | 15 | 1.29 | [0.68, 2.46] |  |
|  | Thailand | 8 | 2 | 0.34 | [0.08, 1.45] |  |
| ECOG performance status^a^ | 0–1 | 78 | 33 | 1 | Reference | 0.240 |
|  | ≥2 | 16 | 9 | 1.56 | [0.74, 3.25] |  |
| Cytogenetic risk^a^ | Standard risk | 49 | 19 | 1 | Reference | 0.099 |
|  | High risk | 14 | 10 | 2.25 | [1.04, 4.88] |  |
|  | Unknown | 41 | 17 | 1.08 | [0.56, 2.09] |  |
| ISS stage^a^ | I | 21 | 8 | 1 | Reference | 0.074 |
|  | II | 23 | 9 | 0.90 | [0.35, 2.36] |  |
|  | III | 34 | 12 | 1.13 | [0.46, 2.77] |  |
|  | Unknown | 26 | 17 | 2.25 | [0.97, 5.25] |  |
| R-ISS stage^a^ | I | 7 | 1 | 1 | Reference | 0.128 |
|  | II | 37 | 15 | 2.00 | [0.26, 15.24] |  |
|  | III | 13 | 3 | 1.17 | [0.12, 11.31] |  |
|  | Unknown | 47 | 27 | 3.38 | [0.46, 24.98] |  |
| Number of prior treatment lines | 1 | 73 | 34 | 1 | Reference | 0.532 |
|  | 2 | 21 | 7 | 0.67 | [0.29, 1.51] |  |
|  | 3 | 10 | 5 | 1.22 | [0.48, 3.12] |  |
| Prior proteasome inhibitor | No | 9 | 2 | 1 | Reference | 0.302 |
|  | Yes | 95 | 44 | 2.11 | [0.51, 8.74] |  |
| Prior immunomodulatory drug | No | 33 | 11 | 1 | Reference | 0.345 |
|  | Yes | 71 | 35 | 1.39 | [0.70, 2.74] |  |
| Relapsed multiple myeloma | No | 9 | 3 | 1 | Reference | 0.352 |
|  | Yes | 95 | 43 | 1.75 | [0.54, 5.66] |  |
| Refractory multiple myeloma | No | 82 | 38 | 1 | Reference | 0.533 |
|  | Yes | 22 | 8 | 0.78 | [0.36, 1.69] |  |
| Bortezomib response  duration^b^ (months) | <6 | 11 | 2 | 1 | Reference | 0.278 |
|  | ≥6 | 70 | 32 | 2.21 | [0.53, 9.23] |  |
|  | <12 | 21 | 7 | 1 | Reference | 0.963 |
|  | ≥12 | 60 | 27 | 1.02 | [0.44, 2.35] |  |
|  | <18 | 31 | 11 | 1 | Reference | 0.677 |
|  | ≥18 | 50 | 23 | 1.17 | [0.57, 2.39] |  |
|  | <24 | 46 | 16 | 1 | Reference | 0.181 |
|  | ≥24 | 35 | 18 | 1.58 | [0.81, 3.11] |  |
| Thalidomide response duration^b^ (months) | <6 | 8 | 3 | 1 | Reference | 0.896 |
|  | ≥6 | 45 | 25 | 1.08 | [0.32, 3.63] |  |
|  | <12 | 14 | 8 | 1 | Reference | 0.331 |
|  | ≥12 | 39 | 20 | 0.66 | [0.29, 1.52] |  |
|  | <18 | 19 | 10 | 1 | Reference | 0.408 |
|  | ≥18 | 34 | 18 | 0.72 | [0.33, 1.57] |  |
|  | <24 | 27 | 13 | 1 | Reference | 0.735 |
|  | ≥24 | 26 | 15 | 1.14 | [0.54, 2.40] |  |
| R-exposure | R-exposed | 25 | 11 | 1 | Reference | 0.202 |
|  | R-naïve | 79 | 35 | 1.60 | [0.78, 3.28] |  |

*CI* confidence interval, *ECOG* Eastern Cooperative Oncology Group, *ISS* International Staging System; *PD* progressive disease, *PR* partial response, *R-ISS* revised International Staging System. ^a^Analysed based on clinical characteristics at multiple myeloma diagnosis; ^b^Bortezomib/thalidomide response duration = (PD date - best response date +1)/30.44 if best response ≥ PR and missing if best response < PR.

**Supplementary Table 2** Univariate Cox regression analysis of factors associated with overall response rate

|  | | N | # of events | Odds Ratio^b^ | 95% CI | P-value |
| --- | --- | --- | --- | --- | --- | --- |
| Age (years) | <65 | 53 | 34 | 1 | Reference | 0.068 |
|  | ≥65 | 51 | 41 | 2.291 | [0.940,5.582] |  |
| Gender | Male | 60 | 42 | 1 | Reference | 0.575 |
|  | Female | 44 | 33 | 1.286 | [0.534,3.093] |  |
| Country of enrolment | Korea | 69 | 55 | 1 | Reference | 0.007* |
|  | Malaysia | 27 | 13 | 0.236 | [0.091,0.615] |  |
|  | Thailand | 8 | 7 | 1.782 | [0.202,15.694] |  |
| ECOG performance status^a^ | 0–1 | 78 | 59 | 1 | Reference | 0.121 |
|  | ≥2 | 16 | 9 | 0.414 | [0.136,1.262] |  |
| Cytogenetic risk^a^ | Standard risk | 49 | 38 | 1 | Reference | 0.471 |
|  | High risk | 14 | 10 | 0.724 | [0.190,2.763] |  |
|  | Unknown | 41 | 27 | 0.558 | [0.220,1.417] |  |
| ISS stage^a^ | I | 21 | 17 | 1 | Reference | 0.458 |
|  | II | 23 | 16 | 0.538 | [0.132,2.193] |  |
|  | III | 34 | 26 | 0.765 | [0.199,2.941] |  |
|  | Unknown | 26 | 16 | 0.376 | [0.098,1.446] |  |
| R-ISS stage^a^ | I | 7 | 5 | 1 | Reference | 0.718 |
|  | II | 37 | 27 | 1.080 | [0.180,6.489] |  |
|  | III | 13 | 11 | 2.200 | [0.237,20.396] |  |
|  | Unknown | 47 | 32 | 0.853 | [0.148,4.914] |  |
| Number of prior lines of therapy | 1 | 73 | 53 | 1 | Reference | 0.982 |
|  | 2 | 21 | 15 | 0.943 | [0.321,2.771] |  |
|  | 3 | 10 | 7 | 0.881 | [0.207,3.742] |  |
| Prior proteasome inhibitor | No | 9 | 6 | 1 | Reference | 0.704 |
|  | Yes | 95 | 69 | 1.327 | [0.309,5.699] |  |
| Prior Immunomodulatory Drug therapy | No | 33 | 26 | 1 | Reference | 0.304 |
|  | Yes | 71 | 49 | 0.600 | [0.226,1.589] |  |
| Relapsed multiple myeloma | No | 9 | 9 | 1 | Reference | - |
|  | Yes | 95 | 66 | NE | [NE, NE] |  |
| Refractory multiple myeloma | No | 82 | 61 | 1 | Reference | 0.321 |
|  | Yes | 22 | 14 | 0.602 | [0.222,1.638] |  |

*CI* confidence interval, *ECOG* Eastern Cooperative Oncology Group, *ISS* International Staging System, *NE*: Not estimable; *R-ISS* revised international staging system.

^a^Analysed based on clinical characteristics at multiple myeloma diagnosis. ^b^ Type 3 test that checks for differences between any pair of sub-categories. *Statistically significant.

**Supplementary Table 3** Univariate Cox regression analysis of factors associated with progression-free survival

|  | | N | # of events | Hazard Ratio | 95% CI | P-value |
| --- | --- | --- | --- | --- | --- | --- |
| Bortezomib response  duration^a^ (months) | <6 | 11 | 2 | 1 | Reference | 0.213 |
|  | ≥6 | 70 | 36 | 2.47 | [0.59, 10.26] |  |
|  | <12 | 21 | 9 | 1 | Reference | 0.823 |
|  | ≥12 | 60 | 29 | 0.92 | [0.43, 1.94] |  |
|  | <18 | 31 | 13 | 1 | Reference | 0.705 |
|  | ≥18 | 50 | 25 | 1.14 | [0.58, 2.23] |  |
|  | <24 | 46 | 20 | 1 | Reference | 0.482 |
|  | ≥24 | 35 | 18 | 1.26 | [0.66, 2.38] |  |
| Thalidomide response duration^a^ (months) | <6 | 8 | 3 | 1 | Reference | 0.803 |
|  | ≥6 | 45 | 23 | 1.17 | [0.35, 3.89] |  |
|  | <12 | 14 | 9 | 1 | Reference | 0.159 |
|  | ≥12 | 39 | 17 | 0.56 | [0.25, 1.26] |  |
|  | <18 | 19 | 12 | 1 | Reference | 0.096 |
|  | ≥18 | 34 | 14 | 0.52 | [0.24, 1.12] |  |
|  | <24 | 27 | 16 | 1 | Reference | 0.215 |
|  | ≥24 | 26 | 10 | 0.61 | [0.27, 1.34] |  |
| R-exposure | R-exposed | 25 | 10 | 1 | Reference | 0.340 |
|  | R-naïve | 79 | 38 | 1.40 | [0.70, 2.82] |  |

*CI* confidence interval, *PD* progressive disease, *PR* partial response.

^a^Bortezomib/thalidomide response duration = (PD date - best response date +1)/30.44 if best response ≥ PR and missing if best response < PR.

**Supplementary Table 4** Univariate Cox regression analysis of factors associated with overall survival

|  | | N | # of events | Hazard Ratio | 95% CI | P-value |
| --- | --- | --- | --- | --- | --- | --- |
| Bortezomib response duration^a^ (months) | <6 | 11 | 2 | 1 | Reference | 0.625 |
|  | ≥6 | 70 | 14 | 0.69 | [0.15, 3.07] |  |
|  | <12 | 21 | 8 | 1 | Reference | 0.007* |
|  | ≥12 | 60 | 8 | 0.26 | [0.10, 0.70] |  |
|  | <18 | 31 | 9 | 1 | Reference | 0.066 |
|  | ≥18 | 50 | 7 | 0.40 | [0.15, 1.06] |  |
|  | <24 | 46 | 11 | 1 | Reference | 0.240 |
|  | ≥24 | 35 | 5 | 0.53 | [0.18, 1.53] |  |
| Thalidomide response duration^a^ (months) | <6 | 8 | 2 | 1 | Reference | 0.469 |
|  | ≥6 | 45 | 10 | 0.56 | [0.12, 2.67] |  |
|  | <12 | 14 | 7 | 1 | Reference | 0.007* |
|  | ≥12 | 39 | 5 | 0.21 | [0.07, 0.66] |  |
|  | <18 | 19 | 9 | 1 | Reference | 0.003* |
|  | ≥18 | 34 | 3 | 0.14 | [0.04, 0.52] |  |
|  | <24 | 27 | 9 | 1 | Reference | 0.068 |
|  | ≥24 | 26 | 3 | 0.29 | [0.08, 1.10] |  |
| R-exposure | R-exposed | 25 | 5 | 1 | Reference | 0.372 |
|  | R-naïve | 79 | 17 | 1.61 | [0.56, 4.60] |  |

*CI* confidence interval, *PD* progressive disease, *PR* partial response

^a^Bortezomib/thalidomide response duration = (PD date - best response date +1)/30.44 if best response ≥ PR and missing if best response < PR
